# The Compass of Commitment: Control Mechanisms Underpinning the Sense of Individual and Joint Commitment

**DOI:** 10.1111/cogs.70136

**Published:** 2025-10-28

**Authors:** Angelica Kaufmann, Martina Fanghella, John Michael

**Affiliations:** ^1^ Cognition in Action Unit, PHILAB, Department of Philosophy, University of Milan

**Keywords:** Commitment, Joint action, Control, Inhibition, Interference suppression, Motivation

## Abstract

The sense of commitment directs us toward our goals, shielding us from distractions and temptations, and thereby facilitates a wide range of cooperative activities and institutions characteristic of our species. Building upon recent research, this paper identifies cognitive, motivational, and social factors that elicit or enhance the sense of commitment. It surveys studies on cognitive and motivational mechanisms, including control mechanisms, that may support the sense of commitment. This research is organised into a framework that enables us to relate these distinct mechanisms to one another. It also allows us to formulate novel hypotheses about how these mechanisms may interact to help us stay on course toward our goals .

## 1 Introduction

Humans exhibit a remarkable ability to remain committed to their goals. From completing individual projects to participating in collective efforts such as scientific research or environmental initiatives, commitment enables persistence in difficult, long‐term goals by shielding them from distractions, temptations, and fluctuations in short‐term interests. How is this achieved?

While much research has explored the cognitive and motivational mechanisms supporting persistence and self‐regulation in individual agency, surprisingly, little attention has been given to how these processes function in joint action (Melis & Semmann, 2010; Sebanz, Bekkering, & Knoblich, 2006; Tomasello, 2009). Yet, the prevalence of joint action presents us with the twin challenges of identifying when and to what extent we should rely upon others to persist in joint actions, and identifying when and to what extent we should resist temptations and distractions, and to persist in making contributions that others are relying on us to make. What mechanisms enable us to solve these challenges? It has been hypothesised that the motivation to remain engaged in joint actions is governed by a sense of commitment, which acts as a sort of compass, directing us to persist and shielding us from tempting alternative options and distractions (Michael, 2022; Michael, Sebanz, & Knoblich, 2016a). According to this hypothesis, the sense of commitment is a disposition to boost persistence in joint action in response to cues indicating that another agent is expecting one to perform one's contribution, and may be relying on this expectation (Dana, Cain, & Dawes, 2006; Heintz, Celse, Giardini, & Max, 2015; MacCormick & Raz, 1972; Michael, 2022; Michael et al., 2016a; Scanlon, 1998; Sugden, 2000). In line with this hypothesis, recent research has identified cues that boost the sense of commitment in joint action. For example, it has been shown that a partner's investment of costs such as effort or time may signal that she expects and is relying upon one to remain engaged and that this in turn motivates one to remain engaged and to persist longer (Székely & Michael, 2018; Chennells & Michael, 2018; Bonalumi, Isella, & Michael, 2019; McEllin & Michael, 2022). Additionally, there is evidence that sensorimotor coordination may also function as a cue indicating that a joint action partner expects and is relying upon one to persist in a joint action, and can accordingly boost persistence in joint action (Michael, Sebanz, & Knoblich, 2016b).

Despite this previous research, the cognitive and motivational mechanisms through which such cues affect the sense of commitment remain unclear. This is because the sense of commitment, which has been conceptualised so far in previous research, is spelt out in functional terms rather than mechanistic terms. As a result, it may involve a range of cognitive and motivational mechanisms, as well as emotions, which fulfil the function of maintaining our valuation of goals and shielding those goals from tempting alternative options and distractions. In the present paper, our starting point is the hypothesis that it is plausible that many of the same underlying mechanisms support commitment in joint action and individual contexts. We, therefore, draw on existing research on individual goal persistence to illuminate the mechanisms that may also underpin commitment in joint action (summarised in Brandstätter & Bernecker, 2022).

To illuminate these mechanisms, we present a framework that bridges insights from individual goal pursuit and social commitment. This framework aims to provide a foundation for developing new hypotheses on the ways these mechanisms interact, potentially reinforcing our ability to persist in pursuing our goals. At its core is a distinction between two forms of commitment, each likely involving distinct mechanisms (Michael, 2022). The first, *gritted‐teeth commitment*, occurs when you experience boredom or distraction, or are otherwise tempted to abandon a goal, but still exert effort to persevere. For example, consider being committed to finishing a swimming session: you might focus intensely on your pace and breathing to ignore the discomfort and even the boredom urging you to stop. The second form, *engaged commitment*, arises when you are so immersed in pursuing a goal that you do not notice temptations or distractions, eliminating the need to force yourself to ignore or resist them. For example, imagine being deeply involved in playing a musical instrument: you are so absorbed in the music that you do not even notice time passing or external noises around you.

We further define gritted‐teeth commitment as persistence through effortful control, requiring the inhibition of distractions and competing motivations. This form of commitment may recruit executive mechanisms such as inhibitory control, working memory, and interference suppression to sustain goal pursuit despite conflicting desires. In contrast, engaged commitment is sustained by mechanisms that reduce the salience of distractions by increasing the motivational and attentional value of the task itself. These mechanisms may include the modulation of attention toward goal‐relevant rewards, the implicit valuation of outcomes through reinforcement learning, and the possible engagement or reward‐related neural systems such as the ventral striatum and medial prefrontal cortex (PFC) (Ryan & Deci, 2017). While engaged commitment does not require the same level of effortful inhibition, it nonetheless involves cognitive processes that stabilise goal‐directed behaviour by enhancing the intrinsic appeal of the task. Thus, both forms of commitment can lead to sustained action over time but appear to rely on distinct underlying mechanisms, one centred on executive resistance, the other on motivational attraction, making conflict less likely.

It is important to emphasise at the outset that this distinction is not directly intended as a hypothesis about the mechanisms underlying the sense of commitment. Rather, it is intended as a heuristic framework, as a rough‐and‐ready guide, or a structuring tool, for our discussion of the mechanisms which may underlie the sense of commitment.^1^ By establishing this distinction upfront, we can better examine how different forms of cognitive control are engaged across contexts. As such, the gritted‐teeth/engaged commitment distinction provides a useful scaffold for exploring the mechanisms behind perseverance, attention, and goal maintenance. Insofar as gritted‐teeth commitment implies persistence in the face of temptations and distractions, it is likely supported by executive function, in particular, inhibitory control and supervisory attentional control. We, therefore, focus on the literature on *cognitive control*. Since engaged commitment, instead, implies a reduction of temptations and distractions, it may involve filtering out irrelevant information and modulating the salience or reward value of goal‐relevant information through motivational processes. Throughout, we reference research on cognitive control, motivation, and reward in both individual and social contexts, given that many of the mechanisms relevant to individual task performance also operate in joint actions.

In addition to identifying mechanisms underpinning gritted‐teeth commitment and engaged commitment, this framework allows us to explore how these mechanisms interact. For example, one hypothesis is that they may be mutually exclusive: either the intrinsic reward value of the task is enhanced (engaged commitment), in which case the need for executive control is reduced, or executive control is enhanced (gritted‐teeth commitment), stabilising task focus and performance despite a reduction in the intrinsic reward value of a task. However, the two may also support one another. For example, gritted‐teeth commitment may work by focusing attention on aspects of a task that are rewarding, leading to an increase in the intrinsic reward value of a task. Indeed, some accounts have proposed that executive control can serve to bias lower‐level processing in working memory (Baddeley, 1986; Christensen, Sutton, & McIlwain, 2016), Conversely, by enhancing the intrinsic reward value of a task, engaged commitment may also lead to the recruitment of executive resources to stabilise or boost performance.

By using this distinction as a structuring tool, we avoid reducing experiences to binary categories. Rather than rigidly separating the two types, we treat them as points on a continuum—acknowledging that tasks may involve both effortful control and intrinsic motivation at different phases.

While we describe *gritted‐teeth commitment* and *engaged commitment* as distinct types for conceptual clarity, we emphasise that these are better understood as idealised ends of a spectrum rather than mutually exclusive categories. This framing serves as a heuristic tool to investigate the cognitive and motivational mechanisms underlying commitment, not as a strict psychological taxonomy. Indeed, as we note throughout the paper (see especially Sections 2 and 4), individuals often shift between these two poles depending on task demands, motivational states, and contextual cues. To our knowledge, no existing literature posits a strict dichotomy in empirical terms, but there is ample evidence supporting distinct neurocognitive profiles associated with control‐heavy, effortful persistence (e.g., Aron, Robbins, & Poldrack, 2014; Braver, 2012; Bunge, Ochsner, Desmond, Glover, & Gabrieli, 2001) and with flow‐like, intrinsically motivated absorption (e.g., Di Domenico & Ryan, 2017; Ott & Nieder, 2019; Ryan & Deci, 2000). Thus, our distinction is intended to generate hypotheses and structure inquiry into how different configurations of control and motivation sustain goal‐directed behaviour over time.

The paper is structured as follows: Section 2 reviews theoretical and empirical research on control mechanisms relevant to commitment. Then, we outline the possible role of control mechanisms—such as inhibition, interference suppression, and anticipation—in stabilising commitment over time. Section 3 discusses the neural underpinnings of cognitive control in scenarios exemplifying commitment. Section 4 explores how motivation interacts with control to achieve persistence. Section 5 contrasts strategies of resistance and avoidance, emphasising how proactive control structures environments to minimise distractions. We then conclude.

## 2 Commitment and control

We examine the role various control mechanisms may play in sustaining and directing the sense of commitment, and outline hypotheses concerning its potential relationships to other mechanisms that support commitment.

Although most empirical studies discussed here focus on control mechanisms outside the context of commitment per se, our goal is to suggest that these mechanisms provide the psychological and neural infrastructure for sustaining commitment, including in joint action. In such contexts, shared expectations, mutual reliance, and coordinated goals likely recruit executive control processes—such as inhibitory control, interference suppression, and anticipation—to align individual actions with collective aims. Thus, the mechanisms that sustain individual commitment can plausibly be extended to explain how individuals manage their roles in joint commitments, especially when motivation fluctuates or distractions arise.

We draw upon a characterisation of control spelt out by Bermúdez (2021): control is the capacity to align actions with commitments despite opposing motivations. As originally observed by Sripada (2020), and later reiterated by Bermúdez, Murray, Chartrand, and Barbosa (2023), researchers diverge in how they define control. Some (e.g., Holton, 2009; Levy, 2017; Sripada, 2020) adopt a “process view,” restricting control to cases where alignment with commitments is achieved through specific mental processes such as willpower or deliberate inhibition. Others (e.g., Mele, 2003, 1987; Heath & Anderson, 2010; Vierkant, 2014; Koi, 2023) endorse a “results view,” which considers any successful alignment of actions with commitments—regardless of the process involved—as an instance of control.

To illustrate the difference, consider the case of Jacob, who intends to adopt a healthier diet. He has two options: he can avoid shopping in stores that sell junk food and decline invitations to dine out with those friends who like junk food to prevent exposure to the temptation of unhealthy eating, or he can shop and dine anywhere he likes but must actively resist the urge to make poor food choices. According to the results view, both methods rely on control, whereas according to the process view, Jacob's avoidance of situations where he may be tempted to buy or eat junk food does not amount to exercising control; he is simply removing the temptation altogether.

Although many conceptions of control can be neatly categorised as process views or as results views, there are also conceptions in the literature that fall in between these two extremes. For example, Coren (2022) focuses on how individuals use inhibitory control and other executive resources to manage desires, but also acknowledges a broader range of control strategies for acting in line with commitments. This view suggests that control is about effectively managing one's actions in ways that resonate with personal values and objectives. This approach provides a starting point for us to next discuss how different types of control interact with the two forms of commitment presented in the introduction.

### 2.1 Types of control

In exploring the dynamic nature of commitment—both toward individual goals and collaborative endeavours—it is essential to consider the different types of control individuals employ to navigate their motivations and tasks. Shepherd (2014) categorises control into four specific types: hot, cold, wide, and narrow. Each appears to play a unique role in navigating the internal terrain of motivation and the external pressures of task environments. We suggest that these forms of control are also central to managing the complexities of commitment. The first two notions are hot and cold control. *Hot control*, often associated with executive control, is described as involving active management of attention and behaviour through mechanisms such as inhibitory control, working memory, and cognitive flexibility (Sel & Shepherd, 2020). It is especially relevant in contexts where motivation is low, but perseverance is necessary—when individuals pursue long‐term goals through sheer willpower despite finding the tasks mundane, difficult, or unrewarding. This kind of control can act as a cognitive rudder, steering actions in alignment with long‐term intentions amid potential distractions (Shepherd, 2015b).

By contrast, cold control is often associated with intrinsic motivation. It tends to involve minimal executive effort and may arise when individuals are naturally drawn to their tasks. In such cases, distractions lose their appeal, and sustained engagement feels effortless. The task itself provides sufficient reward, maintaining commitment without the need for deliberate self‐regulation.

Shepherd (2014) introduces a further distinction between wide and narrow control. *Wide control* allows individuals to modify their behaviour based on shifting circumstances and broader environmental factors. It is crucial for maintaining long‐term goals, as it provides the flexibility and responsiveness needed to adapt to changes that might affect how a person interacts with their tasks or goals (Shepherd, 2022). Conversely, *narrow control* focuses on maintaining a specific and often rigid approach to tasks, making it well‐suited for short‐term goals that require concentrated effort and resistance to distraction. Although Shepherd does not explicitly offer mechanistic explanations of these forms of control, it is plausible that hot and narrow control involve inhibition, working memory, and task‐switching, processes typically associated with PFC activity. In contrast, the mechanisms underlying cold and wide control are less well‐defined. Shepherd's framework provides a way to characterise an agent's ability to exercise control even when opposing motivation exists, but a comprehensive mechanistic explanation is not forthcoming. Yet, a look at cold and wide control is necessary to complete the picture. Cold control, which emerges when an individual's motivations align with their tasks, appears to rely on mechanisms that require minimal executive intervention. Research on intrinsic motivation suggests that the brain's reward systems, particularly the ventral striatum and medial PFC, play a central role in sustaining engagement without exertion (Ryan & Deci, 2017). This contrasts with the dorsolateral PFC's engagement in hot control, where inhibition and working memory are required to sustain focus (Table 1). Cold control may, therefore, be sustained through implicit reinforcement, habit formation, and goal‐integration processes that allow actions to persist without the experience of effortful self‐regulation. Wide control, by contrast, refers to an individual's capacity to dynamically adapt their approach while maintaining long‐term commitments. This form of control is particularly crucial in environments where priorities shift or where multiple concurrent projects require integration. Unlike narrow control, which relies on sustained attention to specific tasks, wide control enables individuals to flexibly allocate cognitive resources and update strategies without losing track of overarching goals. While Shepherd (2014) does not explicitly link wide control to specific cognitive mechanisms, evidence from research on cognitive flexibility suggests that the PFC, particularly the anterior cingulate cortex (ACC) and orbitofrontal regions, may play a key role in adjusting commitments over time (Monsell, 2003). Wide cold control thus emerges when individuals maintain commitment to broad, evolving projects through an effortless sense of engagement rather than deliberate executive effort.

**Table 1:** Types of control. A glossary

Type of control	Definition	Characteristics	Example
Hot control	Involves effortful executive functions like inhibitory control and working memory to maintain focus and resist distractions.	Requires willpower; effortful; used when tasks are unengaging.	Resisting social media distractions while working on a tedious report.
Cold control	Aligns with intrinsic motivation, minimising the need for deliberate cognitive effort by making tasks naturally engaging.	Effortless; intrinsically rewarding; tasks sustain focus naturally.	Getting absorbed in a favourite hobby without external motivation.
Wide control	Allows for flexible adaptation to shifting priorities and environmental changes while maintaining long‐term commitments.	Adaptive; strategic; integrates multiple goals over time.	Managing multiple research projects while shifting between priorities.
Narrow control	Focuses on precision and consistency, applying sustained attention to specific, well‐defined tasks.	Rigid; detailed‐oriented; maintains structured focus.	Focusing intensely on coding a complex experiment to avoid errors.

The concepts of wide and narrow control can be understood as orthogonal to hot and cold control. While wide and narrow control primarily concern the *scope* and *specificity* with which an individual manages tasks and responsibilities, hot and cold control relate to the nature of motivation and the degree of cognitive effort involved.

Wide control is characterised by adaptability and flexibility. It allows individuals to adjust strategies across changing circumstances and to integrate diverse actions over time across different contexts. This form of control is crucial when dynamic adjustment is needed—for example, managing shifting priorities or coordinating long‐term, multifaceted goals. Conversely, narrow control is more focused and precise, often applied in scenarios requiring meticulous attention to detail and a consistent approach. It is employed when tasks need a highly specific, sustained effort and when deviations from established procedures could lead to errors or failures (Shepherd, 2015a). By contrast, hot control involves the exertion of willpower to overcome immediate temptations and distractions, thereby maintaining focus on long‐term goals. It is often activated in situations where there are conflicts between immediate desires and long‐term objectives, requiring a conscious effort to manage competing demands (Shepherd, 2022). Cold control, on the other hand, is less about overcoming distractions and more about aligning actions with intrinsic motivations that do not require immediate, conscious regulation. This type of control is typically in play when tasks are inherently motivating or engaging, and thus, the need for active self‐regulation is minimised.

These two axes—scope (wide/narrow) and motivation (hot/cold)—are best treated as orthogonal, meaning they can combine in different ways depending on the context. Each combination reflects a distinct mode of control that individuals may adopt to navigate specific challenges:

An individual might employ *wide hot control* in situations where they need to adapt to a variety of challenges over time while also managing immediate distractions or temptations. For example, overseeing a long‐term research project with many moving parts needs to adapt strategies frequently while resisting the lure of shortcuts that compromise quality.

*Wide cold control* might be observed where a person engages in a broad range of activities that they find intrinsically rewarding and thus does not require much effort to maintain focus. Consider a researcher working on the development of a series of ideas who might explore different strategies and subjects without needing to consciously push themselves to continue.

*Narrow hot control* is typical in situations that demand precise, focused attention to complete a task correctly under conditions of potential distraction. Imagine our researcher working on a complex design for their experiment, who must focus intensely on the coding without succumbing to external pressures or immediate demands.

*Narrow cold control* would be typical in tasks that are both routine and require precision, but are so well practiced that they are nearly automatic and do not require the resolution of conflict between desires. An example could be a researcher teaching a well‐designed introductory course to their students, where the task demands accuracy but is highly routinised and enjoyable.

These combinations help explain how different control strategies support different kinds of commitment. In the Introduction, we emphasised that gritted teeth and engaged commitment should not be seen as mutually exclusive, but as points along a continuum. People may shift between them depending on the task, context, or evolving interests. For instance, a task initially approached with gritted‐teeth commitment may become more engaging as one develops proficiency or discovers aspects of the task that align with personal interests, thereby shifting toward engaged commitment. Conversely, an initially engaging task may require the application of gritted‐teeth commitment when faced with unexpected challenges or waning interest. These transitions often call upon different forms of control in flexible and context‐sensitive ways.

To illustrate, consider Athena and Jonas, two researchers embarking on a long‐term collaborative project motivated by their shared commitment to academic inquiry. Over several years, they both face the monotonous reality of repetitive data collection and the slow emergence of observable results. This gradual grind begins to wear down Jonas's initial excitement more notably than Athena's. Despite the critical importance of their work, the daily tasks become routine and uninspiring for Jonas. His commitment shifts from passionate engagement to a more obligatory mindset, motivated by the need to complete the project for publication and secure future funding.

This shift can test the stability of their joint commitment. To counter Jonas's growing disenchantment and maintain focus on their shared long‐term goals, Athena and Jonas must rely on what Shepherd calls “hot control.” This involves consciously reaffirming the project's importance, reminding each other of its potential impact, and possibly adjusting their roles to reintroduce some variety and renewed interest. By distributing the more tedious aspects of the research and alternating responsibility for the more engaging components, they can help sustain each other's commitment. This scenario highlights that joint commitment in collaborative projects is not only about sustaining personal motivation but may also be about actively supporting each other in the face of challenges, thereby strengthening their partnership and shared objectives despite the lack of immediate gratification.

In a reversed scenario, Athena and Jonas embark on the same long‐term project not out of genuine enthusiasm, but due to professional obligations and external pressures, such as funding requirements and academic expectations. Their commitment is marked by obligation—a typical example of gritted‐teeth commitment. Early on, neither finds day‐to‐day work engaging. The project requires scrupulous data collection and analysis, often monotonous and slow to yield results. This phase is driven by “narrow hot control,” where both must apply a focused effort to meet clearly defined goals while suppressing distractions and disinterest to adhere closely to a defined process necessary for achieving their long‐term objectives.

As the project progresses, Athena and Jonas begin to discover aspects of their work that resonate more with their interests. They start to see the real‐world impact of their findings or feel intellectually stimulated by the challenges they encounter. As these elements of the work become more apparent, their commitment evolves. The routine data collection starts to connect with broader implications, sparking a genuine interest and engagement that was not present initially.

This shift to engaged commitment may be facilitated by a transition to “wide cold control.” The control exercised by Athena and Jonas becomes less about overcoming distaste for the task and more about harnessing their growing intrinsic motivation to explore and expand their project's scope. Wide cold control enables them to explore new avenues within the project, integrating their tasks with their evolving interests and the emerging opportunities for innovation and discovery in their field. This form of control supports their ability to engage more deeply without the constant need for self‐regulation against boredom, reluctance, or temptations.

Athena and Jonas find that their work becomes more rewarding. They are absorbed by their research, and the effort they put into their tasks feels increasingly worthwhile and fulfilling. This dynamic not only strengthens their partnership but also enhances the quality and creativity of their research. The transformation from narrow hot to wide cold control reflects a deeper shift—from gritted‐teeth commitment to engaged commitment—indicating how evolving circumstances and discoveries within a project can fundamentally reshape the emotional and motivational terrain of collaborative work.

These scenarios illustrate that commitment is not static, but fluid and responsive to both internal states and external conditions. Understanding how different types of control contribute to navigating these shifts provides valuable insight into how individuals can sustain long‐term dedication, adapt to challenges, and manage their motivation effectively over time.

Formally, these narrative examples can be interpreted within our framework as illustrating transitions between different control profiles in joint action. Narrow hot control underpins gritted‐teeth commitment by stabilising persistence in the face of distraction through inhibitory and attentional regulation. Wide cold control underpins engaged commitment by amplifying the intrinsic motivational salience of the task and supporting flexible, exploratory engagement. By situating the scenarios in this way, we highlight how shifts in joint commitment can be described in terms of underlying cognitive mechanisms, rather than only in terms of lived experience.

In addition to the types of control discussed, it is important to consider the hierarchical structure of commitments and the varying types of control employed at different levels of nested projects. Often, individuals manage a series of subprojects that exist within larger overarching projects—each requiring a distinct type of control. For example, while overseeing a long‐term research project (the main goal), subtasks such as organising a workshop or reviewing a colleague's manuscript might demand different control mechanisms. Some tasks may operate in the foreground, necessitating narrow, hot control with focused attention and inhibition, while others might move to the background, relying on wide, cold control where intrinsic motivation or routine allows for more flexible engagement. This layered structure of activity raises important questions: can multiple forms of control operate in parallel, or do they shift dynamically as certain tasks move into focus while others recede into the background? The alternation between foregrounded and backgrounded projects might itself be a form of meta‐control, allowing individuals to fluidly transition between different cognitive and motivational states. Our framework suggests that gritted‐teeth commitment is navigated using a compass of executive or hot control. Contrastingly, engaged commitment may be directed by a compass that aligns with cold control.

We begin with gritted‐teeth commitment, which typifies perseverance in the absence of intrinsic motivation. This form of commitment is typically invoked in the face of tasks that we might find mundane, challenging, or otherwise unengaging, yet recognised as necessary for achieving a longer‐term goal. The cognitive compass we take to guide gritted‐teeth commitment likely involves a suite of processes, including inhibitory control, working memory, and cognitive flexibility. These processes act as the rudder, suppressing distractions and maintaining the course of commitment through the turbulent waters of potential temptations, ensuring that one's actions remain aligned with long‐term objectives, even in the absence of immediate rewards.

In contrast, engaged commitment arises when a task is inherently fulfilling. Here, commitment is not a struggle but a natural extension of one's intrinsic motivations and interests. This form of commitment is directed by a cognitive compass that aligns with a form of intrinsic control, minimising the need for the deliberate exertion of executive control. Engaging with the task is itself rewarding, thereby sustaining commitment without the need for constant vigilance against distractions.

The distinction between wide and narrow control further clarifies how individuals regulate their commitments. Wide control, characterised by its breadth and adaptability, enables individuals to modify their approach based on shifting circumstances and broader environmental factors. This type of control is crucial for maintaining both gritted teeth and engaged commitment, as it allows for flexibility and responsiveness to changes that might affect how a person interacts with their tasks or goals. Conversely, narrow control focuses on maintaining a specific and often rigid approach to tasks, emphasising precision and consistency even in the face of distractions. This form of control is again relevant to both gritted teeth and engaged commitment, where the focus is on overcoming immediate, defined obstacles through concentrated effort and closely managed attention.

This analysis highlights that commitment is not a static state but a dynamic one that can fluctuate due to various internal and external factors. In this context, control may play a pivotal role in stabilising commitment through the operation of mechanisms which enable individuals to adapt to changes, manage their motivations, and align their actions with their goals (Gottfredson & Hirschi, 1990; Matsuela, 2008).

With this in mind, we propose a framework which is structured around three components that aim to explain how control, the sense of commitment, and motivation interact to sustain goal‐directed behaviour. The proposed framework is not defined by the internal distinctions of its components themselves but by how they illuminate the interaction between cognitive control, motivation, and the sense of commitment. In this way, the framework is both integrative and generative—offering concrete conceptual tools for guiding future research and empirically testable prediction.

To clarify how the framework is organised, it is important to note the levels of abstraction at which its components operate. Control mechanisms (such as anticipation, inhibition, and interference suppression, see Section 2.2) are discussed here at the most concrete level, regulating attention and behaviour in real time. We treat the sense of commitment as operating at an intermediate level, linking these control mechanisms to goals and stabilising goal pursuit across time (see Section 3). Motivation is treated as the most abstract component, providing a general orientation for behaviour (e.g., results‐oriented vs. task‐oriented, see Section 4). Although distinguished here for explanatory clarity, these components are dynamically interconnected: control can stabilise motivation, while motivation in turn influences which control strategies are recruited.

In our framework, the sense of commitment is hypothesised to function as the link between control mechanisms and goals: it may stabilise our valuation of goals in the face of distractions and temptations, and may activate the control mechanisms which govern our thoughts and behaviours accordingly. We distinguish two primary forms of commitment: gritted‐teeth commitment and engaged commitment. Gritted‐teeth commitment arises when an individual is pushing through a task out of necessity, often relying on hot control to manage distractions and maintain focus. In contrast, engaged commitment happens when an individual is deeply absorbed in the task, which aligns with cold control and minimises the need for deliberate effort. These two forms of commitment mark opposite ends of a spectrum, and individuals may shift between them on a gradient depending on the task and context.

**Fig. 1:**
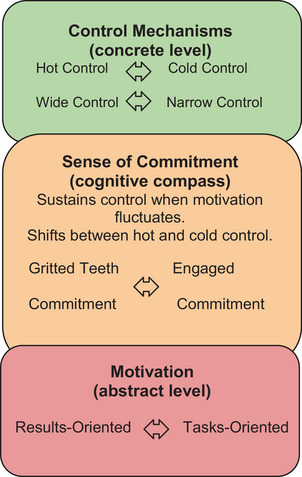
Schematic diagram: control mechanisms, sense of commitment, and motivation.

Finally, motivation, which we will discuss in Section 4, is shaped by two forces: one is “results‐oriented” and typically associated with extrinsic motivations, producing gritted‐teeth commitment, where effort is required to stay focused. The other is “tasks‐oriented,” and typically associated with intrinsic motivation, producing engaged commitment, where the task is naturally enjoyable, requiring little effort to maintain (Fig. 1).

Thus, the framework sketches how control mechanisms might operate and how the sense of commitment may act as a dynamic mediator, influenced by the fluctuating states of motivation that guide whether actions are goal‐driven or task‐driven. Together, these components explain how commitment can maintain control over an agent's actions even when their motivation varies across temporal scales, goals, and tasks.

While the previous section emphasised how different types of control relate to varying forms of commitment, it is equally important to examine the underlying cognitive mechanisms that make such regulation possible.

### 2.2 Cognitive control: Anticipation, inhibition, and interference suppression

Control includes a broad scope of mechanisms that can help to manage competing influences and distractions that may disrupt commitment. These mechanisms include anticipation, inhibition, and interference suppression.

Anticipation refers to the ability to foresee potential distractions or challenges and prepare responses in advance, enabling individuals to preemptively adjust their strategies to stay aligned with their goals (Braver, 2012). For example, a surgeon anticipating complications may prepare backup tools and strategies to quickly address issues as they arise, thereby maintaining focus and ensuring patient safety.

Inhibition is the capacity to suppress responses that may not be conducive to the current goal. It involves the deliberate effort to hold back actions that could derail focus and commitment (Aron et al., 2004; Munakata et al., 2011). For example, inhibition might prevent someone from impulsively checking their phone during an important task.

Interference suppression is the capacity to selectively manage and mitigate the impact of external and internal distractions that interfere with the execution of intended actions (Friedman & Miyake, 2004). This can include preventing actions, both planned and unplanned, from being initiated, as well as the ability to interrupt or modify the pace of an ongoing activity. For example, a student in a noisy environment might use interference suppression to focus on their studies by mentally blocking out the surrounding chatter and concentrating on their reading material.

The mechanisms underpinning interference suppression seem to involve complex cognitive processes that enable an individual to prioritise actions based on their relevance and urgency, thus ensuring alignment with overarching commitments. Interference suppression differs from inhibitory control in that it captures a wider range of strategic adjustments and responses to dynamic and potentially disruptive environments (Nee, Wager, & Jonides, 2007; Bunge, Dudukovic, Thomason, Vaidya, & Gabrieli, 2002).

Anticipation, however, differs from both interference suppression and inhibitory control as it involves the proactive prediction and preparation for future events rather than merely responding to present stimuli or actions. Anticipation requires cognitive processes that enable individuals to foresee potential disruptions or opportunities and adjust their strategies in advance to optimise outcomes. Unlike interference suppression, which deals with immediate distractions, and inhibitory control, which focuses on stopping prepotent responses, anticipation is forward‐looking, emphasising preparatory actions and the allocation of attention to upcoming scenarios. This proactive stance can allow for a more strategic alignment of behaviour with long‐term goals and commitments. Here, we focus on inhibitory control and interference suppression, which appear integral to the broader construct of cognitive control, since much remains to be explored about how exactly they facilitate the suppression of interfering actions, especially in the context of commitment (Botvinick, Braver, Barch, Carter, & Cohen, 2001; Miller & Cohen, 2001).

While these mechanisms play a role in self‐regulation and goal‐directed behaviour, it is important to distinguish cognitive control—a broad category of processes that facilitate goal maintenance—from self‐control, which specifically refers to the ability to override short‐term impulses in favour of long‐term goals (Baumeister, Vohs, & Tice, 2007; Duckworth, Gendler, & Gross, 2016).

Self‐control is often considered a subset of cognitive control that is particularly relevant in contexts requiring resistance to immediate temptations (e.g., resisting the urge to check one's phone while working). However, control mechanisms such as interference suppression and anticipation extend beyond self‐control, as they are also involved in broader processes of flexible goal maintenance, adaptive behaviour, and strategic planning. These processes may support commitment not only by helping individuals regulate impulses but also by allowing them to proactively structure their environments and expectations to facilitate long‐term engagement with their goals (Botvinick & Cohen, 2014).

By distinguishing between self‐control and cognitive control more broadly, we aim to clarify that while self‐control may be crucial for resisting distractions, the mechanisms underlying commitment may not be limited to impulse inhibition. Instead, they include proactive forms of control such as environmental structuring, attentional allocation, and goal maintenance mechanisms, which are central to sustaining commitment in both individual and social contexts.

## 3 Empirical evidence of the mechanisms that may underlie the sense of commitment

We now turn to empirical research that contributes to elucidating the cognitive and neural mechanisms that may underlie the sense of commitment and, in particular, the different types of cognitive control. To clarify the link between cognitive control and the sense of commitment, we propose that commitment—especially in its joint and diachronic forms—requires more than the mere formation of intentions. It demands stabilising intention over time, often in the face of internal or external conflict, and doing so in a way that is sensitive to social expectations and shared plans. This stabilisation process has been argued to rely heavily on specific cognitive control mechanisms—in particular, interference suppression, inhibitory control, and the strategic deployment of proactive versus reactive regulation. While most empirical studies on cognitive control do not frame their findings in terms of commitment, we argue that the mechanisms of control plausibly constitute the neural and psychological infrastructure that may make commitment possible—both individually and jointly. In the following sections, we adopt a structured approach: first outlining the relevant control mechanisms, then reviewing behavioural, neuroimaging, and lesion studies, and finally suggesting how these findings support our framework by corroborating the distinction of commitment strategies reflecting “engaged” and “gritted teeth” commitment.

### 3.1 Neural underpinnings of cognitive control: The possible role of the PFC in sustaining commitment

While not explicitly framed in terms of commitment, some studies provide us with indirect evidence for its neural basis by indicating how control mechanisms are implemented in prefrontal networks differently across development. For example, Bunge et al. (2001) investigate the potential role of the PFC in mediating tasks that require the suppression of interference and the inhibition of inappropriate responses—potentially key components of cognitive control. They investigated developmental differences in brain activation associated with cognitive control in children (ages 8–12) and adults. Using event‐related functional magnetic resonance imaging (fMRI), Bunge and colleagues examined participants during a task combining the Eriksen Flanker task (which requires ignoring flanking stimuli) and a go/no‐go task (which requires inhibiting responses to certain stimuli) (Eriksen and Eriksen, 1974). This design allowed them to probe both interference suppression and response inhibition. The results suggested distinct patterns of brain activation between children and adults. In interference suppression, children engaged the PFC in the hemisphere opposite to that of adults, indicating a developmental shift in lateralisation. For response inhibition, children activated posterior regions rather than the prefrontal areas used by adults. Notably, children did not recruit the right ventrolateral PFC—a region that adults relied on for both types of control.

These findings may delineate a developmental trajectory of cognitive control. As the PFC matures, its functional connectivity strengthens, allowing more efficient coordination with other brain regions to support goal‐directed behaviour. From these results, we infer that this maturation may be associated with a growing capacity for sustained commitment. Children's relative immaturity in PFC activation may explain their greater susceptibility to distraction and difficulty inhibiting inappropriate responses. Maturation over development may reflect the gradual strengthening of the PFC's functional connectivity and its ability to coordinate with other brain regions in the service of goal‐oriented behaviour.

Consequently, understanding the development of cognitive control not only provides insights into commitment as a developmental phenomenon but also emphasises the necessity of early interventions that foster prefrontal regulation to strengthen goal‐oriented adherence.

To provide a broader perspective on the neural basis of commitment‐related control mechanisms, we now consider the dual mechanisms underlying interference control within working memory.

Building on the Dual Mechanisms of Control model (De Pisapia & Braver, 2006; Braver, 2012), Burgess and Braver (2010) explored how interference expectancy and fluid intelligence (gF) influence control strategies, distinguishing them between proactive and reactive. This distinction is central to understanding how individuals adjust their cognitive efforts to meet situational demands and sustain commitments.

Proactive control mechanisms are especially relevant for maintaining long‐term commitments, as they involve preemptive adjustments in attention and resource allocation before interference occurs. In contrast, reactive control functions as a compensatory mechanism, correcting deviations from goal‐directed behaviour only after disruptions arise.

According to this model, proactive and reactive control strategies are implemented in two different neural circuits. Proactive control is likely linked to continuous and/or anticipatory activation of the lateral PFC, indicating the ongoing maintenance of task objectives. This sustained goal maintenance provides a top‐down influence that aids in the processing of anticipated high‐demand cognitive tasks. Notably, increased proactive control has been correlated with greater perseverance in long‐term goal pursuits (Braver, Gray, & Burgess, 2007; Dosenbach, Fair, Cohen, Schlaggar, & Petersen, 2008). Conversely, reactive control is characterised by temporary activation of the lateral PFC, alongside a broader network involving other brain regions. This short‐lived activation might signify a bottom‐up reactivation of task goals, triggered either by detecting interference (e.g., involving conflict monitoring regions like the ACC) or by associative and episodic connections (possibly involving posterior cortical or medial temporal lobe regions). Neuroimaging evidence supports this distinction, demonstrating that proactive control is associated with sustained dopaminergic (DA) activity in the PFC, whereas reactive control relies on transient responses mediated by the ACC (Braver, 2012; Levy, 2011). Additionally, the two control mechanisms should vary in their engagement of the DA system. Sustaining inputs in the PFC necessitates a phasic DA‐mediated gating signal present when contextual cues are provided. Without this gating signal, the PFC can only be activated transiently (Braver, 2012).

In Burgess and Braver (2010), the expectancy of interference within task blocks is manipulated to observe how anticipation influences the deployment of proactive versus reactive control strategies. The findings suggest that individuals with higher levels of fluid intelligence (gF) tend to prefer, and more effectively utilise proactive control. This preference underscores proactive control's resource efficiency and reduced cognitive tax over time compared to reactive control, which may be more demanding and less efficient as it deals with interference post‐hoc. This preference is particularly relevant in commitment contexts, where individuals with stronger proactive control mechanisms are better at sustaining long‐term goal engagement without requiring constant reactive adjustments.

Neuroimaging results further illustrate this distinction. Increased activity in the bilateral PFC during high interference expectancy scenarios highlights the engagement of proactive control mechanisms (Braver et al., 2007; Dosenbach et al., 2008). In contrast, activity patterns in the left ventrolateral PFC under low interference expectancy are indicative of reactive control's engagement, pointing to its role in directly handling and mitigating interference once it arises (Aron et al., 2004; Levy, 2011).

The evidence supports the idea that together, proactive and reactive control constitute complementary strategies for regulating behaviour. Their interplay enables individuals to align actions with goals—either by anticipating and preventing distractions or by correcting deviations when they occur. This dual mechanism is fundamental to stabilising commitment and maintaining cognitive performance under varying demands.

To complement these findings, electrophysiological studies offer insights into the temporal dynamics of cognitive control. In particular, the electroencephalogram (EEG) measure of readiness potential (RP) provides a window into how motor preparation and inhibition unfold over time. RP research illustrates how cognitive and physiological mechanisms cooperate to support different forms of commitment, adding temporal precision to the spatial resolution offered by fMRI.

### 3.2 Voluntary action: RP as a marker of inhibitory control

When acting, individuals sometimes have to inhibit motor plans that are inconsistent with their goals. When individuals are “gritting their teeth” to remain committed—when one exerts considerable effort to stay on course despite internal impulses or distractions—this struggle involves not only psychological effort but also physiological processes. Specifically, it requires the formation and inhibition of motor plans that may contradict the agent's intended course of action.

Empirical research on the neural basis of voluntary action and intentionality described a cortical signal, called *readiness potential* (RP) or *Bereitschaftspotential* (BP), associated with the initiation of voluntary movements. First discovered by Kornhuber and Deecke (1965), RP has been characterised as a slow, negative electrical potential mainly detected over the supplementary motor area (SMA) that begins to build up in the brain before a person consciously decides to move. The role of RP gained prominence through Libet's experiment (Libet, Gleason, Wright, & Pearl, 1983). In this study, participants were asked to spontaneously perform a movement, while their EEG and electromyogram activity was measured. Participants also reported the moment they first felt the urge to move (the so‐called W‐time). Strikingly, the RP began several hundred milliseconds before movement onset and consistently preceded the W‐time by about 200 ms. Libet concluded that conscious will may not initiate the RP, as the brain had already begun preparing the movement before the participant reported any conscious decision. Importantly, it has been pointed out that we could veto our brain's unconscious decisions in the period between when we became aware of our intention to move and the movement itself; this veto power is commonly referred to as “free won't” (Ramachandran, 1998). According to this interpretation, voluntary control may not lie in initiating actions, but might lie in stopping them. A recent study used RP to estimate the point of no return in vetoing self‐initiated movements to be about 200 ms before movement onset (Schultze‐Kraft et al., 2016), roughly the same time that Libet thought the veto window opened. However, the evidence on “free won't” is not free from criticism (Filevich, Kühn, & Haggard, 2013). Interestingly, it has been suggested that early and late RP might be associated with two distinct processes, interference suppression and response inhibition, and that these two processes may mature at different developmental stages (Bryce et al., 2011). This interpretation is consistent with Schurger's two‐stage model of the RP, suggesting that a signal can lead to movement when it coincides in time with a stochastic fluctuation that pushes the system over the threshold for movement, either in an early or late stage of motor preparation (Schurger, Sitt, & Dehaene, 2021, 2012).

It is plausible that these mechanisms might be implicated in maintaining the sense of commitment by avoiding conflicting temptations. It is likely that early inhibition of RP (i.e., interference suppression), or no inhibition at all (as conflicting actions might be filtered out before the movement arises), might mainly characterize strategies associated with engaged commitment, while response inhibition, associated with vetoing the motor plan, would be associated with gritted‐teeth commitment. In contrast, exceeding the point of no return to inhibit an action (Schultze‐Kraft et al., 2016) might be associated with failing to resist the temptation to defect and, therefore, breaking the commitment.

Understanding the neural basis of inhibition of inconsistent motor plans provides additional insights into the cognitive and neural processes that underlie the two types of commitment. It highlights the dynamic nature of cognitive control, where the brain must continuously manage and suppress competing actions to maintain alignment with long‐term goals. Future research may clarify the potential role of neural circuits underlying cognitive control, and in particular, inhibitory control, in maintaining the sense of commitment and disentangling between engaged and gritted‐teeth strategies (Table 2).

**Table 2:** Empirical literature on cognitive control and potential roles in sustaining commitment

Mechanisms	Neural evidence	Potential role in sustaining commitment
Interference suppression	PFC activation (Bunge et al., 2001)	Enhances resistance to distractions/temptations
Proactive control	Sustained PFC activity (Braver, 2012)	Support long‐term commitment strategies
Reactive control	ACC and transient PFC activity (Levy, 2011)	Facilitates recovery from lapses in commitment
Response inhibition	RP dynamics (Schultze‐Kraft et al., 2016)	Determines success in suppressing conflicting impulses
Readiness potential (RP)	SMA activation before voluntary action (Libet et al., 1983)	A neural marker of cognitive conflict in commitment?

While cognitive control may be crucial for resisting or avoiding the temptation to break a commitment—a distinction explored further in Section 5—motivation may also play a pivotal role. It energises and directs action, helping maintain focus despite the lure of conflicting alternatives. In the next section, we turn to the motivational states and processes that plausibly drive goal‐directed behaviour and support sustained commitment.

## 4 Control as a motivation stabiliser

This section focuses on the mechanisms through which individuals or groups may sustain their dedication to goals amidst fluctuating motivations and external challenges.

Werner and Milyavskaya (2019) distinguish between “want‐to” motivations and “have‐to” motivations. This differentiation may be pivotal in understanding the dynamics of commitment: while “have‐to” motivations are driven by external pressures—such as monetary incentives or the avoidance of negative outcomes like punishment, or emotional discomfort such as guilt—“want‐to” motivations stem from inherent interest and personal values. According to this distinction, how individuals frame their motivations can significantly affect their performance and the persistence of their commitments.

Bermùdez (2021) further explores how individuals can reframe their motivations from “have‐to” to “want‐to” through skilled control and practical reasoning. This ability to reinterpret the underlying reasons for a task can transform a commitment driven by obligation into one sustained by genuine interest. This capacity for reframing, mediated by executive functions, highlights what may be the role of control in shaping motivation sustainability.

While there is some overlap between this distinction and the one we propose between engaged and gritted‐teeth commitment, they should not be conflated. Want‐to and have‐to motivations describe the sources of motivation, while the engaged versus gritted‐teeth distinction refers to the form commitment takes over time and how individuals sustain goal pursuit. Specifically, engaged commitment can be facilitated by want‐to motivations but is not identical to them; rather, engaged commitment is characterised by a lack of perceived effort or resistance, where individuals remain immersed in an activity without needing to suppress distractions or alternative goals. Similarly, gritted‐teeth commitment may frequently align with have‐to motivations but is distinct in that it involves active cognitive control mechanisms that enable persistence despite competing motivational pulls.

To situate this distinction between want‐to and have‐to motivations, it is important to acknowledge the broader research on intrinsic and extrinsic motivation (e.g., Atkinson, 1964; Hunt, 1965; Koch, 1956; Young, 1961, Calder & Staw, 1975). Deci and Ryan (2000) define intrinsic motivation as doing something because it is inherently interesting or enjoyable, and extrinsic motivation as doing something because it leads to a separable outcome. Extensive studies, such as those summarised by Di Domenico and Ryan (2017), highlight the neurological substrates underlying intrinsic and extrinsic motivation, indicating that intrinsic motivation is strongly associated with activity in the ventral striatum and medial PFC, while extrinsic motivation more often involves engagement of lateral prefrontal and parietal circuits. This research provides a more systematic account of how these motivational states interact with self‐regulation and commitment, reinforcing the idea that control mechanisms can either facilitate intrinsic motivation or compensate for its absence when extrinsic pressures are dominant.

Engaged commitment, being often sustained by intrinsic motivation, has, therefore, a hedonic aspect, as it involves both the pursuit of future goals and the enjoyment of the present task. This dual aspect is crucial for maintaining motivation over time, as it provides both immediate satisfaction and long‐term fulfilment. The hedonic element of engaged commitment could serve as a psychological marker for motivation stabilisation, as the intrinsic pleasure derived from the activity itself can help individuals persist even when external challenges arise. This emphasises the importance of finding or cultivating an inherent interest in tasks, which not only improves immediate engagement but also strengthens the overall commitment to long‐term goals.

While control may not directly engage the motivational system, it may indirectly stabilise motivation by helping us avoid temptations and distractions. Thus, although engaged commitment is usually characterised by intrinsic motivation and does not involve the use of executive resources, it may depend indirectly upon forms of control which are driven by extrinsic motivation, typical of gritted‐teeth commitment. This is in line with our hypothesis that engaged and gritted‐teeth commitment are not dichotomous, but are sustained by partly overlapping cognitive and motivational mechanisms.

While the above framework effectively captures individual commitment processes, group commitments introduce additional dynamics that can alter motivation stabilisation. In groups, commitments often emerge not only from individual interests but also from collective goals, shared values, and social dynamics. Group members may experience “want‐to” motivations through a shared vision or collective enthusiasm, which can enhance individual commitment through social reinforcement and shared responsibility.

However, group commitments can also be sustained by “have‐to” motivations, such as peer pressure, organisational culture, or formal obligations, which might not align with individual members’ values or interests. These external pressures can sometimes lead to conflicts between individual and group motivations, requiring more complex forms of control and negotiation to maintain commitment.

Engaged commitment in groups, therefore, not only seems to have a hedonic aspect as in individual contexts, but could also involve a harmonisation of individual desires with collective aspirations. This dual aspect may be crucial for maintaining motivation over time in group settings, providing both immediate social cohesion and long‐term goal fulfilment. The hedonic element of engaged commitment in groups can serve as a psychological marker for motivation stabilisation, as the intrinsic pleasure derived from collaborative activity itself can help members persist even when external challenges arise.

Thus, control in group settings does not directly engage the motivational system but does indirectly stabilise motivation by helping members to align individual interests with group goals, avoid group‐specific temptations and distractions, and navigate interpersonal dynamics.

Control mechanisms in individuals and groups may function as stabilisers in two key ways. First, according to Ott and Nieder (2019), control, particularly through the mechanisms of inhibitory control and interference suppression, plays a crucial role in maintaining focus and consistency. Their research highlights that executive functions, mediated by the PFC, are thought to be integral in managing distractions and competing motivations, thereby, we could say, supporting sustained commitment. Specifically, they discuss how the PFC regulates cognitive control through DA modulation, which enhances the ability to suppress irrelevant stimuli and sustain attention on goal‐relevant tasks (Ott & Nieder, 2019).

To see how, recall the concept of interference suppression discussed in the previous sections. This is a crucial mechanism by which control facilitates the maintenance of engaged commitment. To recap, interference suppression refers to the cognitive and behavioural processes through which individuals mitigate or eliminate the impact of distractions, competing motivations, or adverse conditions on their pursuit of goals. This mechanism could be essential for understanding how control operates as a motivation stabiliser by enabling individuals to focus on their commitments, suppress conflicting desires or distractions, and adapt to changing circumstances.

Second, Hosenbocus and Chahal (2012) emphasise the importance of the executive system in regulating behaviours through a central coordinating system, which may be essential for goal‐directed tasks and self‐regulation. They note that dopamine is a key neurotransmitter in this system, implicating its role in various psychiatric disorders where executive function deficits are prevalent, such as attention deficit hyperactivity disorder and autism spectrum disorder. Their review suggests that children with these disorders often struggle with executive functions such as planning, organising, and completing tasks, which are critical for sustained commitment. The use of medications that target the DA system has been shown to improve these executive functions, thereby enhancing the ability to maintain consistent effort and commitment (Hosenbocus & Chahal, 2012).

To summarise, control may function as a stabiliser of motivation in two distinct but complementary ways: first, by suppressing distractions and competing impulses, particularly through top‐down inhibition and interference suppression; second, by regulating motivational salience via DA pathways, enabling individuals to prioritise goal‐relevant cues and sustain behavioural focus.

Moreover, control can be reactive—involving effortful suppression of temptation—or proactive, where individuals shape their environment to avoid such temptations in the first place. This distinction maps onto the difference between gritted‐teeth commitment, which draws heavily on active resistance, and engaged commitment, which often benefits from prior environmental structuring that minimises the need for suppression.

These dynamics also appear in group settings, where motivation stabilisation involves aligning personal desires with collective aims. In such contexts, engaged commitment can emerge from shared purpose and social reinforcement, while gritted‐teeth commitment may be sustained through formal roles, obligations, or peer pressure. Control mechanisms help individuals navigate the potential friction between personal and group goals, manage social distractions, and maintain alignment with collective trajectories.

In sum, control mechanisms stabilise motivation not by replacing it, but by protecting it—buffering it from distraction, reframing it through reasoning, and scaffolding it with proactive environmental adjustments. Executive functions, particularly interference suppression and inhibitory control, may thus be essential for sustaining both individual and collective commitment in the long term.

We have emphasised the centrality of control mechanisms through which the sense of commitment enables us to resist temptations and distractions and to persist in acting toward our goals. But what if, instead of struggling to resist temptations and distractions, we can simply avoid them?

## 5 Control as avoidance and resistance

A final dimension of our framework concerns the distinction between resisting temptations once they arise and avoiding them in advance. While earlier sections outlined how different forms of control support commitment (e.g., hot vs. cold, narrow vs. wide), here we suggest how these forms can be applied to characterise two complementary strategies: reactive resistance and proactive avoidance. This distinction highlights not just how agents persist in the face of conflict, but how they can also conserve resources by shaping environments and routines to reduce conflict before it emerges.

The role of control in commitment may be twofold. Control could be exercised reactively, to resist temptations as they arise, or proactively, to avoid encountering them altogether.

Nanay (2020) argues that avoiding temptations is not only more efficient than resisting them (Hofmann, Baumeister, Förster, & Vohs, 2012; Ent, Baumeister, & Tice, 2015; Duckworth et al., 2016; deRidder, Lensvelt‐Mulders, Finkenauer, Stok, & Baumeister, 2012), but that the ability to do so depends on the degree of mental fragmentation (Schroeder, 2004; Holton, 2009; Berridge & Zajonc, 1991; Berridge & Robinson, 1995, 2003; Berridge, Robinson, & Aldridge, 2009). A fragmented mind, characterised by inconsistencies and conflicts within one's mental setup, makes resisting temptations more difficult because it requires significant mental effort to manage these inconsistencies. By contrast, a less fragmented mind can more easily sidestep potential conflicts through strategic avoidance.

At first glance, Nanay's emphasis on avoidance may seem to challenge the idea that resistance may be essential to commitment. However, we argue that it broadens rather than undermines the concept of control. Here is why.

Avoidance is not an alternative to control but a proactive expression of it, achieved by strategically managing one's environment to preemptively minimise the occurrence of temptations. This broader understanding of control includes planning and foresight, which can be critical aspects of navigating one's commitments successfully (Tangney et al., 2004). This method does not negate the role of control; it simply shifts the focus from a reactive to a proactive stance, which is often more efficient and sustainable. Avoidance strategies can be seen as aligning one's environment with one's long‐term desires and commitments. By reducing the presence or impact of conflicting options, one may ensure that the compass of commitment points steadily toward the intended direction without frequent recalibrations. This alignment is a form of cold and wide control (see Section 2), ensuring that commitments are pursued in a supportive context.

For example, when someone removes junk food from their kitchen, this is not merely a behavioral decision—it involves goal‐based filtering (identifying unhealthy snacks as inconsistent with one's health goals), working memory updating (holding the goal of healthy eating active during the planning phase), and inhibitory downregulation (resisting impulses to “keep them just in case”). Such forms of cold control also include implementation intentions—“if‐then” action plans that automate avoidance behaviour. These intentions are cognitively encoded rules (e.g., “If I feel bored at work, then I'll go for a walk instead of checking social media”) that reduce cognitive load by delegating control to context‐sensitive triggers.

Conversely, a person navigating a career transition while maintaining a family life and personal health regime requires wide control to adapt strategies in one domain without destabilising others. Such a person must remain sensitive to shifts in goal salience and recalibrate priorities accordingly. They may modify routines and restructure aspects of their environment—for example, relocating closer to work to reclaim time for personal activities—to avoid the temptation to neglect family. In doing so, they also draw on identity‐level commitments: by conceiving themselves as, say, a responsible parent or a committed researcher, they create motivational coherence across domains. These identity anchors may support stability and resilience in the face of new temptations or pressures.

Both cold and wide control may be integral to maintaining a smooth and effective commitment to one's goals. Together, these forms of control provide a comprehensive framework for navigating personal and professional commitments with greater ease and less friction.

Koi (2023) offers additional lenses through which we can understand the strategies of managing temptations and distractions, by emphasising that avoidance and resistance are not mutually exclusive but complementary strategies. In this view, hot control can be understood as sustaining resistance in the face of temptation, while cold control often supports avoidance by aligning one's environment and habits with intrinsic motivations. For instance, to go back to the example mentioned in Section 2, describing the situation of two researchers engaged in a long‐term project, Athena might continue to work through complex analytical problems despite the allure of easier, less demanding tasks, and Jonas might choose a quiet, secluded office to work, removing potential distractions to foster a deep, intrinsic engagement with the research process (cold control).

Integrating Koi's functional aspects of self‐control into our understanding of hot and cold control provides a more holistic view of how individuals can effectively navigate their commitments. By recognising the plausible roles of both direct resistance and environmental alignment, we can appreciate the varied strategies through which individuals achieve their goals, whether through the active exertion of willpower or by crafting conducive settings that align with deeper motivations.

To sum up, control is not only about resisting temptations but also about strategically managing one's environment and internal capacities to align actions with long‐term commitments and core values. By balancing resistance and avoidance, individuals can conserve cognitive resources, adapt to changing circumstances, and sustain their commitments more effectively. In this way, control is not merely a tool for managing commitment but a cornerstone of sustained engagement.

## 6 Conclusion

Commitment is essential not only for pursuing individual goals but also for fostering human sociality and enabling joint actions where people share long‐term objectives. Control is likely a crucial component in the navigation of individual and collective goals we commit to, acting as a compass that directs us through potential distractions and adversities. In this paper, we have discussed the mechanisms that may underlie our ability to maintain focus and dedication over time.

We suggested that gritted‐teeth commitment and engaged commitment represent two ends of a spectrum, each involving distinct cognitive and behavioural processes. Gritted‐teeth commitment relies heavily on executive or hot control, utilising inhibitory control, working memory, and cognitive flexibility to resist temptations and distractions. Engaged commitment, on the other hand, often thrives on intrinsic motivations or cold control, where the inherent enjoyment of the task sustains focus and effort without the need for constant self‐regulation. Moreover, the concepts of wide and narrow control further refine our understanding of how individuals adapt their strategies based on task demands and environmental changes. Wide control provides the adaptability needed for long‐term goals, while narrow control may help ensure precision and consistency in the face of immediate challenges.

We also examined the possible mechanisms of cognitive control—anticipation, inhibition, and interference suppression. Anticipation allows individuals to prepare for potential distractions; inhibition helps suppress responses that could derail focus and commitment; and interference suppression enables the filtering of irrelevant information, ensuring alignment with overarching commitments. These mechanisms may be integral to maintaining both gritted teeth and engaged commitment.

At the neural level, the PFC has been proposed as a candidate hub for executive control functions that may support commitment, based on converging evidence from neuroimaging and lesion studies. Additionally, we proposed that RP—a slow, negative electrical potential detected over the SMA that begins to build up before a person consciously decides to move—could serve as a neural marker for different inhibitory control mechanisms underlying commitment when opposing motivations arise. Indeed, electrophysiological activity associated with RP may reveal inhibitory control mechanisms and voluntary action, thus suggesting how the brain can prepare and suppress motor plans that are inconsistent with one's goals, aiding both gritted teeth and engaged forms of commitment.

We further explored how motivational orientation—specifically the distinction between “want‐to” and “have‐to” motivations (Werner & Milyavskaya, 2019)—shapes the character and stability of commitment. Engaged commitment, driven by intrinsic interest and individual values, encompasses both future‐oriented goals and the immediate pleasure of task engagement, serving as a robust stabiliser of motivation. The hedonic aspect of engaged commitment, involving the pursuit of future goals and the enjoyment of the present task, is crucial for maintaining motivation over time. This dual aspect provides both immediate satisfaction and long‐term fulfilment, which helps individuals persist even when external challenges arise.

The strategies of resistance and avoidance highlight a different set of approaches to maintaining commitment. Resistance involves the active effort to suppress temptations and distractions, often relying on hot control. Avoidance, however, emphasises preemptively structuring one's environment to minimise the presence of temptations, aligning with cold and wide control. This proactive strategy reduces the cognitive load required to maintain commitment, allowing individuals to focus more on their goals. Ultimately, the interplay between different forms of commitment and control highlights the dynamic nature of our motivational systems. By fostering environments and strategies that align with intrinsic motivations, we can enhance our ability to sustain commitment, navigate challenges, and achieve our goals. Importantly, our framework offers novel implications for the study of joint commitment and joint action. Our approach suggests that the same control mechanisms—particularly inhibitory control, interference suppression, and strategic avoidance—may operate within social interactions where mutual reliance and expectation are key. In joint action contexts, gritted‐teeth commitment may manifest when agents persist in their role despite discomfort, while engaged commitment may emerge when social synchronisation or shared goals increase intrinsic motivation.

Future research could test whether joint action involving high coordination, mutual responsiveness, or effort investment leads to measurable differences in control‐related neural activation. For instance, increased dorsolateral PFC engagement may be associated with gritted‐teeth commitment, or heightened ventral striatal activity could characterise engaged commitment during a joint action task.

Moreover, we hypothesise that social cues such as being observed, receiving feedback, or anticipating partner disappointment may recruit cold control mechanisms by increasing the salience of shared goals and facilitating proactive avoidance of distractions. Studies using dual‐EEG hyperscanning, for instance, could track whether synchrony between partners is associated with reduced demand for inhibitory control and enhanced motivation stabilisation.

Additionally, our framework predicts that misalignment in motivational states (e.g., one partner driven by “have‐to” motives and the other by “want‐to”) may challenge sustained joint commitment and lead to breakdowns unless wide control mechanisms—such as metacognitive monitoring and goal reconfiguration—are activated to realign intentions. This hypothesis can be tested experimentally by manipulating partner motivation and tracking commitment over time in joint tasks.

In sum, our framework bridges individual and joint contexts by suggesting how control mechanisms stabilise commitment across both. We hope future empirical work will operationalise the types of commitment and control we propose, allowing the field to explore not only how joint commitments are sustained—but also how they can be repaired, realigned, or intensified.

## Note

1 This differentiation shares some elements with existing psychological constructs. Engaged commitment is compatible with Csikszentmihalyi's (1990) concept of flow, in which individuals experience deep focus and intrinsic motivation while performing a task. Whereas, gritted‐teeth commitment is compatible with the idea of prevention focus in Higgins’ (2012) regulatory focus theory, where individuals persist to avoid negative outcomes, in contrast to promotion focus, which aligns more closely with engagement and intrinsic motivation. Our framework, however, refines these ideas by specifically examining how different control mechanisms interact with commitment in both individual and joint action contexts.
